# The Role of the Rodent Insula in Anxiety

**DOI:** 10.3389/fphys.2019.00330

**Published:** 2019-03-29

**Authors:** Maxs Méndez-Ruette, Sergio Linsambarth, Rodrigo Moraga-Amaro, Daisy Quintana-Donoso, Luis Méndez, Giovanni Tamburini, Francisca Cornejo, Rodrigo F. Torres, Jimmy Stehberg

**Affiliations:** Laboratorio de Neurobiología, Instituto de Ciencias Biomédicas, Facultad de Medicina y Facultad de Ciencias de la Vida, Universidad Andrés Bello, Santiago, Chile

**Keywords:** insula, insular cortex, anxiety, elevated plus maze, CNQX, Bicuculline, rat

## Abstract

The human insula has been consistently reported to be overactivated in all anxiety disorders, activation which has been suggested to be proportional to the level of anxiety and shown to decrease with effective anxiolytic treatment. Nonetheless, studies evaluating the direct role of the insula in anxiety are lacking. Here, we set out to investigate the role of the rodent insula in anxiety by either inactivating different insular regions via microinjections of glutamatergic AMPA receptor antagonist CNQX or activating them by microinjection of GABA receptor antagonist bicuculline in rats, before measuring anxiety-like behavior using the elevated plus maze. Inactivation of caudal and medial insular regions induced anxiogenic effects, while their activation induced anxiolytic effects. In contrast, inactivation of more rostral areas induced anxiolytic effects and their activation, anxiogenic effects. These results suggest that the insula in the rat has a role in the modulation of anxiety-like behavior in rats, showing regional differences; rostral regions have an anxiogenic role, while medial and caudal regions have an anxiolytic role, with a transition area around bregma +0.5. The present study suggests that the insula has a direct role in anxiety.

## Introduction

Anxiety may be defined as an intensified fear or avoidance in response to objects or situations in the absence of true danger (Shin and Liberzon, 2010), and may be operationally defined as an emotional response to potential unidentified threats, being characterized by sustained arousal, vigilance, worry and apprehension that results in specific patterns of defensive behaviors and concomitant autonomic responses (Tovote et al., 2015). It is a normal physiological response commonly found after or in anticipation of stressful experiences, but can also appear frequently as a symptom in patients suffering from neurological and psychiatric disorders, as well as in chronic disease (Nagai et al., 2007; Brooks and Stein, 2015). Anxiety is present in daily life, being among the main symptoms arising from occupational stress, and associated to metabolic syndrome (Tang et al., 2017), obesity (Gariepy et al., 2010) and binge eating (Rosenbaum and White, 2015). Disorders consisting of exacerbated anxiety responses are known as anxiety-related disorders and include, according to the DSM-V, PTSD, GAD, Phobias, panic disorder, social phobia, agoraphobia, OCD, and specific phobias (American Psychiatric Association, 2013). Anxiety-related disorders are the most common disorders in psychiatry, affecting at least 18% of Americans (Kessler et al., 2005) and 7.3% Worldwide (Baxter et al., 2013). Despite the high prevalence of anxiety, the neurobiological basis of anxiety is only beginning to be unveiled.

Several brain regions have been associated to anxiety in humans and rodents, among which, the amygdala has shown to be pivotal in orchestrating behavioral responses to both fear and anxiety (Tovote et al., 2015). Other brain areas that have been associated to anxiety include the bed nucleus of the stria terminalis, hippocampus, septum, paraventricular nucleus of the hypothalamus, ventral tegmental area and locus coeruleus, among others (reviewed in Linsambarth et al., 2017).

The IC, also known as the insula in humans, is a brain region that has been associated to anxiety, albeit being far less studied than the above brain regions. Neuroimaging studies suggest that the insula plays a critical role in the pathophysiology of anxiety-related disorders (Rauch et al., 1997; Damsa et al., 2009). In fact, increased insular activity has been reported in patients suffering from generalized anxiety (Hoehn-Saric et al., 2004), panic disorders (Uchida et al., 2008), phobias (Hoehn-Saric et al., 2004; Goossens et al., 2007; Wendt et al., 2008), OCD (Rauch et al., 1997; Lazaro et al., 2008) and PTSD (Rauch et al., 1997; Lindauer et al., 2008). In all the above disorders, insular activity decreased in response to effective treatment (Hoehn-Saric et al., 2004; Goossens et al., 2007; Lazaro et al., 2008). Despite the vast evidence demonstrating a relationship between anxiety-related disorders and insular activity in humans, the role of the insula in anxiety *per se* remains unclear. A handful of studies in humans suggest that the insula may have a role in anxiety; anxiety may modulate insula recruitment (Dennis et al., 2011); the severity of anxiety may be positively correlated with central amygdala-insula functional connectivity (Wang et al., 2002); and lorazepam induces a dose-dependent reduction in the activation of both the amygdala and insula during emotion processing (Paulus and Stein, 2006). Hence, insular activation may be considered a common feature among anxiety-related disorders, and it may have a role in anxiety *per se*, in concert with amygdala activation.

The insula or IC is located deep within the temporal lobe in humans and surrounds the rhinal fissure in rodents. It is generally associated with pain (Jasmin et al., 2004; Lu et al., 2016), somatosensory (Stephani et al., 2011), gustatory (Kosar et al., 1986; Stehberg and Simon, 2011; Stehberg et al., 2011), viscerosensory (Saper, 1982), auditory (Bieser, 1998) and olfactory (Soudry et al., 2011) functions, and direct IC stimulation causes somatosensory, viscerosensory, motor, vestibular, auditory and speech effects in humans (Nguyen et al., 2009). The insula has also a role in drug addiction (Contreras et al., 2007; Droutman et al., 2015), motor control, language (Mutschler et al., 2009), memory (Bermudez-Rattoni et al., 2005; Fornari et al., 2012, Fornari et al., 2016), emotions (Craig, 2009) and awareness (Kurth et al., 2010). The insula has hence been described as an integrating center, linking information from diverse functional systems, into one subjective image of “our world” (Kurth et al., 2010).

The insula receives interoceptive information, including muscular and visceral sensations and is involved in the regulation of hunger and thirst (Craig, 2002; Berlucchi and Aglioti, 2010). Given its interoceptive inputs, Paulus and Stein proposed that “the insula is crucial in determining the difference between the interoceptive sensation expected from a stimulus and the prediction of its outcome, represented as a prediction signal in the anterior IC” (Paulus and Stein, 2006). They proposed that a difference between the two may initiate an anxiety state and the affective, cognitive and behavioral components that characterize anxiety may be a consequence of this altered prediction signal (Paulus and Stein, 2006).

Understanding the role of the insula in anxiety requires studies in animal models, yet research for the role of the insula in anxiety in animals is currently limited to two studies in rats. Li et al. (2014) reported that muscarinic cholinergic manipulation in the IC of rats modulates anxiety-like behavior in the EPM, which is one of the most accepted models for measuring anxiety-like behavior in rodents (for reviews on the different animal models to measure anxiety-like behaviors see Cryan and Sweeney, 2011; Kumar et al., 2013). In another study, Rojas et al. (2015) showed that insular adrenergic activity modulates arousal-induced increases in neophobia, or hyponeophagia, which is another anxiety-like behavior used to measure anxiety in rats (Dulawa and Hen, 2005). Interestingly, the two studies performed pharmacological manipulations at different regions of the insula. A more systematic evaluation of the role of the different regions of the IC in anxiety is lacking. Here, we microinfused the AMPA receptor antagonist CNQX and the GABA receptor antagonist BMI into 4 sub-regions along the rostro-caudal axis of the IC in order to inhibit or activate the IC, respectively, and used the EPM to measure anxiety-like behavior in rats. Our results suggest a direct, rostro-caudal differential involvement of the IC in anxiety.

## Materials and Methods

### Animals

Male Sprague-Dawley rats weighting 250–300 g were housed individually in a 12-h light-dark cycle and kept in their homecages throughout the study. Animals were removed briefly for stereotaxic surgery, microinjection and behavioral assays. Water and food were provided *ad libitum.* All procedures involving animals were approved by the bioethical Committee of the Universidad Andres Bello, Acta 11/2016 and were in accordance with the United States National Institutes of Health guide lines.

### Cannula Implantation

Rats were chronically implanted with chronic cannulas as previously described (Rojas et al., 2015). Briefly, rats were anesthetized by a combination of ketamine/xylazine/acepromazine (5, 5, and 5 ml/kg, respectively) and mounted in a stereotaxic apparatus. They were given a subcutaneous lidocaine injection under the scalp and their skull was surgically exposed. 21-gauge stainless steel guide cannulas were bilaterally implanted and positioned 1 mm above the IC, according to stereotaxic coordinates relative to bregma aiming at 4 different regions of the insula along its rostro-caudal axis: Rostral agranular (raIC; AP +2.8, ML ±4.0, DV −4.2) (Jasmin et al., 2004); gustatory (gIC; AP +1.2, ML ± 5.6, DV −6.7) (Moraga-Amaro et al., 2014; Rojas et al., 2015); primary interoceptive posterior (pIC; AP −0.5, ML ±5.5, DV −6.0) (Casanova et al., 2016); caudal (cIC; AP −2.3, ML ±6.4, DV −5.4) (Benison et al., 2011). Cannulas were secured by four screws and fixed to the skull using acrylic dental cement. A stylus was inserted into the guide cannula. After surgery, animals received a subcutaneous injection of meloxicam (Pro-vet, Chile; 0.6 mg/kg) and a bacitracin/neomycin dermal ointment (Laboratorio Chile) was applied over the surgical area. A post-surgery recovery period of 7 days was given before behavioral evaluation began and a 3-min daily handling session was carried out during the last 3 days of the recovery period to habituate the animals to microinjection-associated handling stress.

### Microinfusion

A 25-gauge injection cannula was inserted into the guide cannula protruding 1 mm beyond its tip, into the target area. Bilateral microinfusion of either CNQX (6-cyano-7-nitroquinoxaline-2,3-dione, 0.89 μg/μL/0.5 μL/hemisphere; SIGMA), Bicuculline methiodide (BMI) (7.34 ng/μL/0.5 μL/hemisphere; SIGMA) or their respective vehicles (2% DMSO in saline (0.9% NaCl) for CNQX and saline alone for BMI) was performed using an injection cannula that were connected by PE20 tubing to Hamilton micro-syringes driven by a digital microinfusion pump. Microinjections were delivered at a rate of 0.25 μl/min and a diffusion time of 2 min was given by holding the injection cannula in place. The microinfused volume was selected to diffuse into dorso-ventral layers of the IC, allowing to focus on possible rostro-caudal differences (Foilb et al., 2016). Microinfusion was performed 15 min (for CNQX) and 10 min (for BMI) prior to the EPM evaluation in accordance to previous studies (Sanders and Shekhar, 1995; Mathis et al., 2015). Microinjections were performed prior and not during the behavioral task in order to avoid behavioral abnormalities resulting from microinjection-related discomfort.

The number of animals used per group is shown in Table 1.

**Table 1:** Number of animals included in the analysis for each injection.

Injection location	Drug	N° animals
raIC	CNQX	6
	Vehicle (2% DMSO+saline)	5
	BMI	6
	Vehicle (saline)	5
gIC	CNQX	15
	Vehicle (2% DMSO+saline)	16
	BMI	6
	Vehicle (saline)	5
pIC	CNQX	11
	Vehicle (2% DMSO+saline)	11
	BMI	8
	Vehicle (saline)	6
cIC	CNQX	5
	Vehicle (2% DMSO+saline)	9
(openfield)	CNQX	8
(openfield)	Vehicle (2% DMSO+saline)	9
	BMI	7
	Vehicle (saline)	9

### Elevated Plus Maze (EPM)

A single 5-min exposure to the EPM was performed as described elsewhere (Walf and Frye, 2007) with slight modifications. The EPM consisted of four arms, each with a length of 60 cm and 15 cm width. Two opposing arms were open, and the other two were enclosed by 15 cm high black acrylic walls. The maze was standing 75 cm above the floor and was dimly illuminated. After microinjection, rats were placed in the central zone of the maze facing an open arm and allowed 5 min of free exploration. Before each test, the arena was cleaned with 70% ethanol. The evaluation was video recorded for offline analysis, and the time spent on the open arms was manually registered by a blinded investigator. Spontaneous locomotor activity was evaluated by the number of transitions, which represents the number of entrances and exits to and from any of the EPM arms. Time spent in the open arms is presented as percentage of the total evaluation time ± standard error of the mean (SEM). Results were compared by a two tailed Student’s *t*-test when comparing the infused drug and its vehicle for each location and by one-way ANOVA with a Bonferroni *post hoc* test when multiple comparisons were required. A value of *p* < 0.05 was considered statistically significant. In the case of finding significant differences in transitions using EPM, these were corroborated in the open field.

### Open Field

Experiments that showed differences in transitions using the EPM were repeated using the openfield. In this test, microinjected animals were placed at the center of a plexiglas rectangular box (60 × 40 × 40 cm) and allowed to explore for 5 min. The behavior was recorded digitally for subsequent off-line analysis by a blinded investigator, using 10 × 10 cm virtual squares on the floor of the OF. The number of transitions (crossings of the virtual squares) were measured and compared between groups.

### Histology

Immediately after behavioral testing, animals were euthanized and perfused with phosphate buffered saline (PBS) followed by 4% PFA dissolved in PBS. Brains were kept for 48 h in a 30% sucrose solution and then sectioned in a cryostat. For histological analysis, Nissl staining was performed and analyzed using light microscopy. Histological exclusion criteria included cannula misplacement, tissue damage extending beyond the tip of the injection or guide cannulas (as assessed by cellular debris, lack of neuronal somata or gliosis). Animals that met any of these criteria were excluded from further analysis. The Paxinos and Watson rat brain atlas in stereotaxic coordinates was used as primary reference to locate the injection sites (Paxinos and Watson, 2007). The histological analysis was performed blinded to the results obtained from the behavioral evaluation.

## Results

**FIGURE 1:**
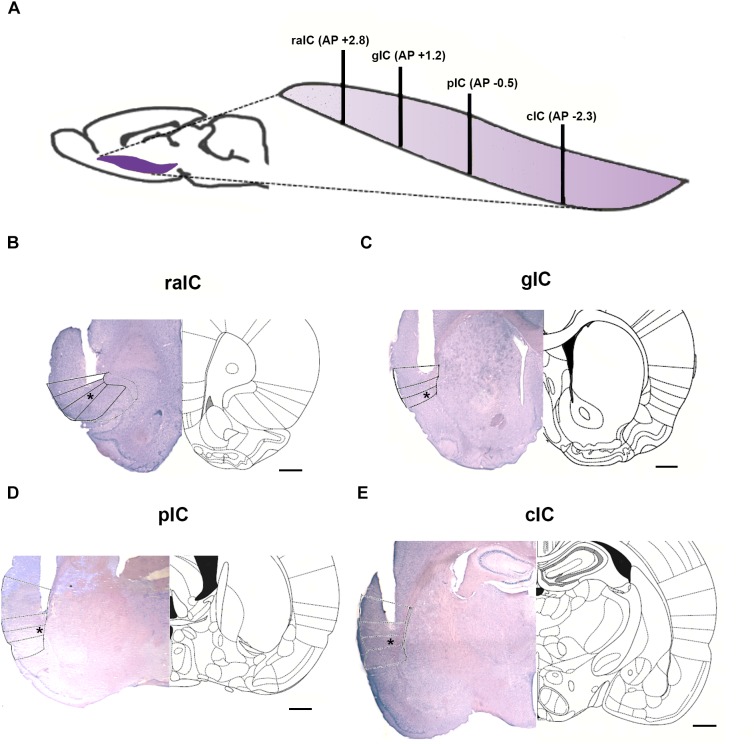
The four IC sub regions studied. **(A)** Schematic rostro-caudal localization of the four IC sub-regions microinjected. Anteroposterior (AP) coordinates relative to bregma are shown for each sub-region in brackets. Black lines show the localization of the respective coronal sections shown in **B–E**. **(B–E)** Representative coronal sections of a microinjection for each insular subregion (left) and its corresponding scheme from Paxinos and Watson (2007) atlas; injection site is shown with an asterisk. **(B)** raIC, rostral agranular insular cortex; **(C)** gIC, gustatory agranular insular cortex; **(D)** pIC, primary interoceptive insular cortex; **(E)** cIC, caudal insular cortex. Scale bar: 0.5 mm.

To evaluate the role of the IC in anxiety, 4 sub-regions along the rostro-caudal axis of the IC were selected for drug microinfusion. The regions selected were: Rostral Agranular Insular Cortex (raIC); gIC; Primary Interoceptive Posterior Insular Cortex (pIC) and Caudal Insular Cortex (cIC). The rostro-caudal location of the 4 sub-regions of the IC is depicted in Figure 1A and representative photomicrographs of cannula placement for each sub-region are shown in Figure 1B–E. Nissl staining was used to evaluate cannula placement and histological damage. Animals that had damage larger than the guide cannula or did not have the tip of the injection cannula at the target area bilaterally were excluded from the analysis.

**FIGURE 2:**
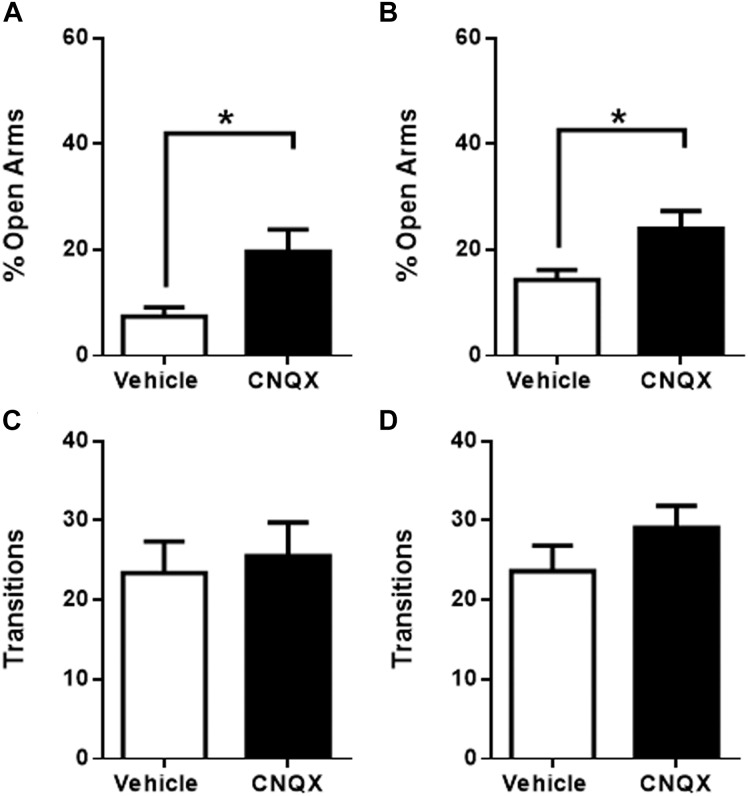
CNQX microinfusion into the rostral sub-regions induces anxiolytic effects in the elevated plus maze (EPM). **(A)** Percentage of time spent in the open arms when vehicle (white bars, *n* = 5) or CNQX (black bars, *n* = 6) was microinjected into raIC; **(B)** Percentage of time spent in the open arms when vehicle (white bars, *n* = 16) or CNQX (black bars, *n* = 15) was microinjected into gIC. Total transitions during the EPM evaluation for rats microinjected into raIC **(C)** or gIC **(D)**. Columns represent the mean ± SEM. ^∗^*p* < 0.05.

Micro-infusion of CNQX into raIC caused a significant increase in time spent in the open arms when compared to vehicle microinjected rats (7 ± 2%, *p* < 0.05, *n* = 5 for vehicle and 20 ± 4%, *n* = 6 for CNQX, *p* < 0.05) (Figure 2A), suggesting an anxiolytic effect. Similarly, CNQX infusion into gIC resulted in decreased anxiety-like behavior (14 ± 2%, *p* < 0.05, *n* = 16 for vehicle and 24 ± 3%, *n* = 15 for CNQX, *p* < 0.05) (Figure 2B). No significant differences in the number of transitions were observed for either sub-region (raIC: 23 ± 4, *n* = 5 for vehicle and 26 ± 4, *n* = 6 for CNQX. gIC: 24 ± 3, *n* = 16 for vehicle and 29 ± 3, *n* = 15 for CNQX; *p* > 0.05) (Figure 2C–D), suggesting that changes in the time spent in the open arms are not due to locomotor abnormalities, sedation or decreased exploration. Given that inactivation of both the raIC and gIC by CNQX induced a decrease in anxiety as measured in the EPM, the role of both rostral regions of the insula may be considered putatively anxiogenic.

**FIGURE 3:**
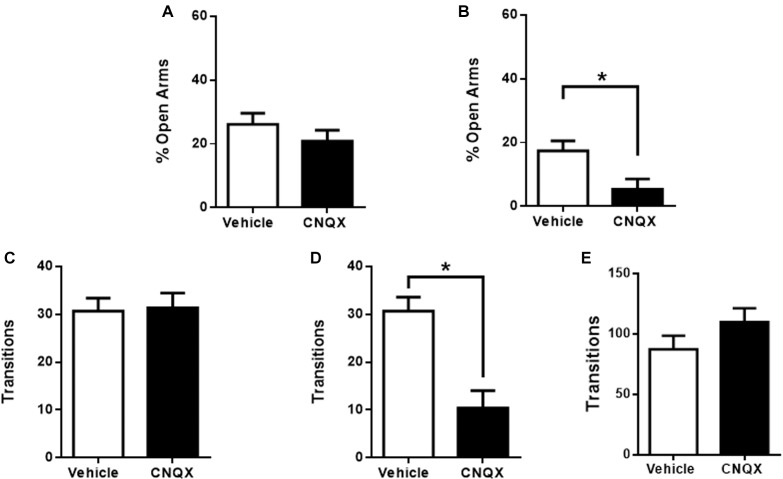
CNQX microinfusion into the caudal sub-regions of the IC induces anxiogenic effects in the EPM. **(A)** Percentage of time spent in the open arms when vehicle (white bars, *n* = 11) or CNQX (black bars, *n* = 11) was microinjected into pIC; **(B)** Percentage of time spent in the open arms when vehicle (white bars, *n* = 9) or CNQX (black bars, *n* = 5) was microinjected into cIC. Total transitions during the EPM evaluation for the same rats microinjected into pIC **(C)** or cIC. **(D)**. **(E)** Effect of CNQX microinjected into the cIC in locomotion (transitions) in the open field **(E)**. Columns represent the mean ± SEM. ^∗^*p* < 0.05.

Microinjection of CNQX into the pIC had no significant effects on anxiety but showed a tendency to decrease the time spent in the open arms when compared to vehicle microinjected rats, which did not reach significance (26 ± 4%, *n* = 11 for vehicle and 21 ± 3%, *n* = 11 for CNQX, *p* > 0.05) (Figure 3A). Vehicle and CNQX microinjected rats showed no differences in transitions for pIC, discarding locomotor abnormalities or changes in exploratory behavior (pIC: 31 ± 3, *n* = 11 for vehicle and 31 ± 3, *n* = 11 for CNQX; *p* > 0.05) (Figure 3C).

The inactivation of the cIC significantly decreased the time spent in the open arms when compared to vehicle microinjected rats (17 ± 3%, *n* = 9 for vehicle and 5 ± 3%, *n* = 5 for CNQX, *p* < 0.05) (Figure 3B). However, CNQX microinjected into the cIC induced a significant decrease in transitions (cIC: 31 ± 3, *n* = 9 for vehicle and 10 ± 4, *n* = 5 for CNQX; *p* < 0.05; Figure 3D). To determine whether this difference is due to motor abnormalities, locomotion was measured in the open field and no differences were found when compared to veh injected rats, suggesting that the difference in transitions found in the EPM was not due to decreased locomotion, suggesting possible increased anxiety (cIC: 87 ± 11, *n* = 8 for vehicle and 110 ± 11, *n* = 9 for CNQX; *p* > 0.05; Figure 3E).

**FIGURE 4:**
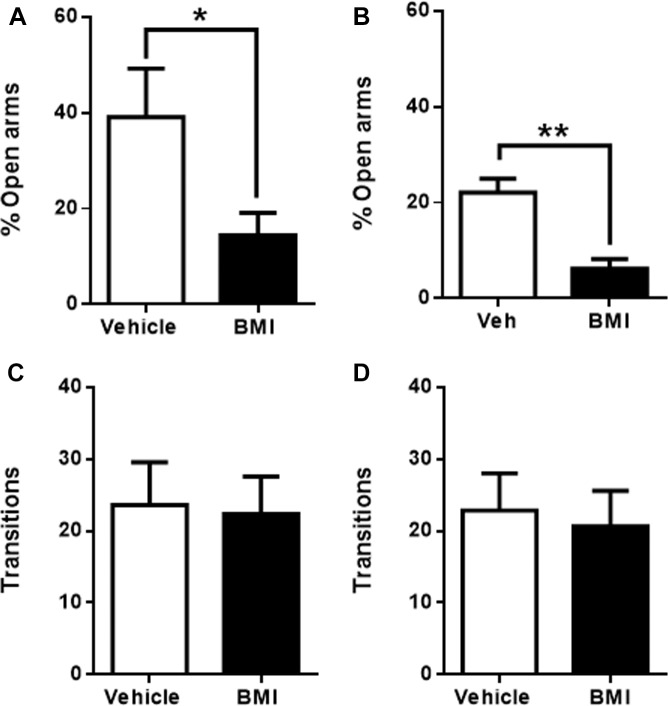
Bicuculline microinfusion into the rostral sub-regions in the EPM induces anxiogenic effects. **(A)** Percentage of time spent in the open arms when vehicle (white bars, *n* = 5) or BMI (black bars, *n* = 6) was microinjected into raIC; **(B)** Percentage of time spent in the open arms when vehicle (white bars, *n* = 5) or BMI (black bars, *n* = 6) was microinjected into gIC. Total transitions during the EPM evaluation for the same rats microinjected into pIC **(C)** or cIC **(D)**. Columns represent the mean ± SEM. ^∗^*p* < 0.05 and ^∗∗^*p* < 0.01.

To corroborate the above results, the four insular regions were now activated by the microinjection of GABA receptor antagonist bicuculline (BMI). Congruent with an anxiogenic role for raIC, BMI microinjection into the raIC induced anxiogenic-like effects, producing a significant decrease in the time spent in the open arms when compared to vehicle injected rats (39 ± 10%, *n* = 5 for vehicle and 14 ± 5%, *n* = 6 for BMI, *p* < 0.05; Figure 4A). Likewise, BMI microinjection into the gIC also induced a significant decrease in the time spent in the open arms when compared to its corresponding vehicle (18 ± 4%, *n* = 6 for vehicle and 5 ± 2%, *n* = 6 for BMI, *p* < 0.01; Figure 4B). No differences in the number of transitions were observed for either sub-region (raIC: 24 ± 6, *n* = 5 for vehicle and 22 ± 12, *n* = 6 for CNQX. gIC: 23 ± 5, *n* = 6 for vehicle and 21 ± 5, *n* = 6 for CNQX; *p* > 0.05) (Figure 4C,D), suggesting that changes in the time spent in the open arms are not due to locomotor abnormalities, sedation or decreased exploration. In summary, inactivation with CNQX induced anxiolytic effects, while activation with BMI induced anxiogenic effects in both the raIC and gIC regions of the insula, suggesting an anxiogenic role for the rostral portion of the IC.

**FIGURE 5:**
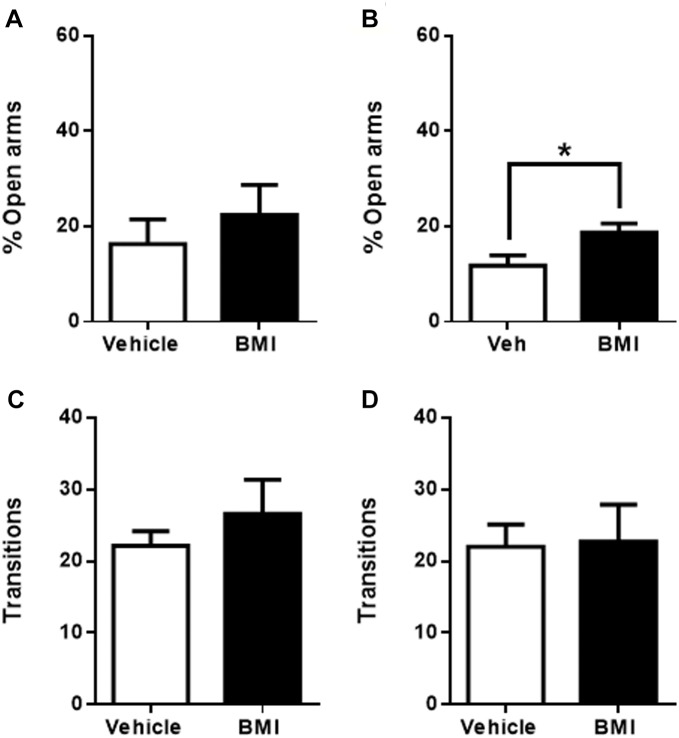
Bicuculline microinfusion into the caudal sub-regions induces anxiolytic effects in the EPM. **(A)** Percentage of time spent in the open arms when vehicle (white bars, *n* = 6) or BMI (black bars, *n* = 8) was microinjected into pIC; **(B)** Percentage of time spent in the open arms when vehicle (white bars, *n* = 9) or BMI (black bars, *n* = 7) was microinjected into cIC. Total transitions during the EPM evaluation for the same rats microinjected into pIC **(C)** or cIC **(D)**. Columns represent the mean ± SEM. ^∗^*p* < 0.5.

Bicuculline microinjection into the pIC induced an increase in the time spent in the open arms (16 ± 5%, *n* = 7 for vehicle and 22 ± 6%, *n* = 8 for BMI, *p* > 0.05), which – as with CNQX – again did not reach significance (Figure 5A).

In line with the results obtained from CNQX microinjection into the most caudal region of the insula, BMI microinjection into the cIC induced anxiolytic effects (Figure 5B), producing a significant increase in the time spent in the open arms when compared to vehicle injected rats (12 ± 2%, *n* = 9 for vehicle and 19 ± 2%, *n* = 7 for BMI, *p* < 0.05). There were no differences in the number of transitions in either group (pIC or cIC) when compared to their respective controls (cIC: 22 ± 3, *n* = 9 for vehicle and 23 ± 5, *n* = 7 for BMI; pIC: 22 ± 2, *n* = 7 for vehicle and 27 ± 5, *n* = 8 for BMI; *p* > 0.05) (Figure 5C,D), suggesting that changes in the time spent in the open arms were not due to locomotor abnormalities, sedation or increased exploration. Given that the activation of the cIC induced a decrease in anxiety and its inactivation induced an increase in anxiety as measured in the EPM, the role of the most caudal region of the insula (cIC) may be considered putatively anxiolytic.

**FIGURE 6:**
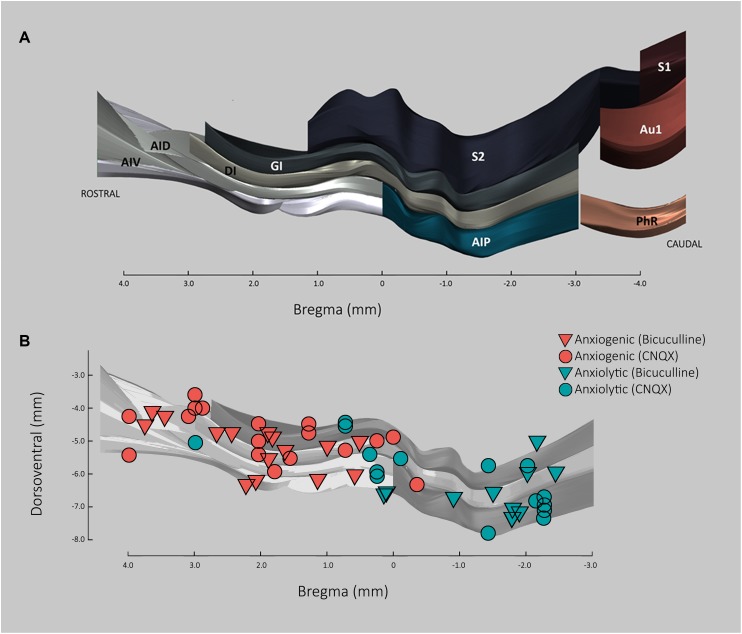
Anxiolytic and anxiogenic roles of the insular cortex based on location and effect of CNQX and Bicuculline microinjections. **(A)** 3D representation of insula cortex based on Paxinos and Watson (2007), showing dorso-ventral subdivisions along the rostral axis in respect to bregma. AID, agranular insular cortex dorsal part; AIP, agranular insular cortex posterior part; AIV, agranular insular cortex ventral part; Au1, primary auditory cortex; DI, dysgranular insular cortex; GI, granular insular cortex; PhR, perirhinal cortex; S1, primary somatosensory cortex; S2, secondary somatosensory cortex. **(B)** Anxiolytic and anxiogenic roles along insular cortex based on CNQX and Bicuculline microinfusions. Each triangle/circle represent an injection case (circles, CNQX; triangles, Bicuculline). The anxiolytic/anxiogenic classification was qualitative and defined as the role of that particular location in anxiety. The role in anxiety for each injection site was estimated based on the difference between the time in the open arms of each case and the average of the respective control. Given that CNQX is an inhibitor, final values for CNQX were multiplied by –1. Final values <0 were considered anxiogenic and >0, anxiolytic.

To determine whether the lack of significance in the anxiogenic effects of CNQX and BMI in the pIC may be due to its possible location within a transition zone between anxiogenic and anxiolytic regions of the insula, each microinjection location for CNQX and BMI was plotted along the rostro-caudal axis and the ventro-dorsal subdivisions of the insula (see Figure 6A), together with the anxiogenic or anxiolytic role of that region based on the difference between the anxiety-like behavior of each animal and the average of the corresponding control group. As can be seen in Figure 6B, the transition area in which both anxiogenic and anxiolytic roles for the insula can be found after microinjections of both CNQX and BMI, runs along approximately bregma +0.5 and −0.5. As pIC microinjections fell within the caudal end of this transition area (around bregma −0.5 mm), the effects of pharmacologically modulating the activity of this area showed mixed results with a tendency for an anxiolytic role, making it difficult to reach significance.

The present results suggest that rostral regions of the insula may have an anxiogenic role, while caudal regions may have an anxiolytic role, with a transition region between bregma +0.5 and −0.5. As can be seen in Figure 6B, no dorso-ventral differences in the effects of microinjections were observed, except possibly for the dysgranular IC, which appears to remain anxiogenic for most of the transition zone.

## Discussion

The sub-regions selected for the present work, have previously been used for studies regarding learning and memory (Hasanein and Sharifi, 2015), drug addiction (Casanova et al., 2016), taste function (Stehberg et al., 2011; Moraga-Amaro et al., 2014; Rojas et al., 2015) and pain (Jasmin et al., 2004; Benison et al., 2011). However, their direct role in anxiety had not been explored. It has been suggested that the anterior insula contributes to the perception of interoceptive sensations associated to danger or threat, possibly playing a crucial role in anxiety (Paulus and Stein, 2006). However, delimitations of insular regions in the rostrocaudal axis are not as straightforward as in the dorso-ventral axis (Maffei et al., 2012). We used the AMPA receptor antagonist CNQX to study the role of IC in anxiety and to address whether functional differences could be observed along the rostro-caudal axis of the IC by inactivating the IC in different regions along its rostro-caudal axis. CNQX disturbs glutamatergic transmission and hence neuronal activation (Wiltgen et al., 2010), providing an inversed insight into the role of a brain region in behavior. CNQX microinfusion into raIC and gIC caused an increase in the time spent in the open arms, suggesting an anxiogenic role for these rostral IC sub-regions. These results were corroborated with GABA receptor antagonist BMI, which showed that activation of both regions induces anxiogenic effects in the rat. These results support a pivotal role for the anterior insular regions in anxiety and help to understand the neuroimaging studies evidencing higher IC activity in patients suffering from anxiety-related disorders (Nagai et al., 2007; Brooks and Stein, 2015).

A previous study determined a role for IC muscarinic receptors in anxiety (Li et al., 2014). It is important to note that this work considered IC coordinates at the level of the most caudal sub-region studied here, cIC. Concordantly, CNQX microinjection into the cIC decreased the time spent in the open arms, suggesting an anxiolytic role for the most caudal region of the insula, which was corroborated by BMI injections.

Perhaps the most parsimonious explanation for this regional difference along the rostro-caudal axis of the IC is the existence of different neuronal populations in the rostral and caudal portions, with their respective anxiogenic and anxiolytic outputs. This may be similar to the bed nucleus of the stria terminalis, which has shown to consist of different cell populations with differential anxiogenic and anxiolytic effects (Kim et al., 2013). In the case of the insula, the rostral regions may include anxiogenic neuronal populations while the most caudal regions may show anxiolytic neuronal populations, which may coexist within the transition area described in the present report. Further studies will be required to understand the anatomical correlates that subserve the differential role of the insula along its rostro-caudal axis.

Another alternative explanation may be related to the flow of interoceptive information within the IC. An internal, caudal to rostral relay of interoceptive information within the IC has been previously proposed (Cauda et al., 2012; Droutman et al., 2015). Therefore, it is plausible that CNQX or BMI microinjections into caudal regions (e.g., cIC) impairs caudal to rostral connectivity within the insula, changing activity in the rostral sub-regions. Based on this idea, CNQX microinjected into the more caudal regions of the insula would be expected to induce anxiolytic effects, since the behavioral output would be commanded by the decreased input into the anxiogenic rostral regions. Further studies must be performed to determine the role of caudal to rostral connectivity within the insula in anxiety.

Notwithstanding, the present results show a direct involvement of the IC in anxiety. Furthermore, we show that the IC involvement in anxiety is not homogenous along its rostro-caudal axis. The location of the insula within the brain circuitry of anxiety is currently unknown. In that respect, the amygdala and insula interact closely with each other and appear to share many functional aspects (for a review see Moraga-Amaro and Stehberg, 2012). This is particularly important as the amygdala is pivotal in orchestrating behavioral responses to fear and anxiety (Tovote et al., 2015). Both insula and amygdala have been shown to increase their activation during emotion processing in normal volunteers (Paulus et al., 2005), anxiety-prone subjects (Stein et al., 2007) and phobia patients (Wright et al., 2003), activation that can be reduced by anxiolytics (Paulus et al., 2005). Increased functional connectivity between the insula and the amygdala has also been reported in untreated GAD patients (Liu et al., 2015), and during acute stress following a traumatic event (Osuch et al., 2008). Increased functional connectivity between the amygdala and the insula has also been positively correlated with the severity of anxiety symptoms (Wang et al., 2002).

There are extensive amygdala-insula connections in rodents (Allen et al., 1991). Studies in rodents have also shown projections bringing somatosensory and pain information to the amygdala from somatosensory cortical areas running across the insula (Shi and Cassell, 1998). Furthermore, glutamate injections into the basolateral amygdala induce decreases in spontaneous discharges at the gIC (Hanamori, 2009). Moreover, it has been suggested that the insula may receive discomfort signals from the body, and then integrates and sends the signals to the amygdala (Davis, 1990; Baur et al., 2013). The insula and the amygdala are also reciprocally connected with the ventral tegmental area, which releases dopamine and its activation induces anxiolytic effects (Jennings et al., 2013). Connections between the insula and other anxiety relevant brain regions have also been reported in rodents, including the lateral hypothalamic area (Allen et al., 1991), bed nucleus of the stria terminalis (Saper, 1982) and nucleus accumbens (Wright and Groenewegen, 1996; Jaramillo et al., 2018). Based on the present study, the insula appears to be critical for anxiety. Hence, its position with respect to other regions within the anxiety-related brain circuitry is an issue that warrants the need for further investigation.

In our study, saline and 2% DMSO (in saline) were used as vehicles for BMI and CNQX, respectively. The effects in the EPM for each injection at any location was compared to the effects of the microinjection of its respective vehicle in that same location. It must be noted, however, that there was high variability in the baseline values after vehicle microinjections, which could reflect differences in the level of anxiety among groups of animals or individuals. However, the possibility that the vehicle injections may have affected anxiety cannot be readily ruled out, in which case, the drugs could have altered the effects of the vehicle (either prevented or enhanced them), rather than induced effects in anxiety by themselves. Hence, care must be taken when interpreting the present results. Further research will be required to determine the possible effects of vehicle injections into the IC in anxiety.

A final point that deserves to be discussed is related to whether the role of the insula in the rat can be directly translated to the human insula. In this respect, several studies have suggested vast similarities between the human and the rodent insula, including their functional organization (reviewed in Moraga-Amaro and Stehberg, 2012). However, on the basis of mediodorsal thalamic afferents, the dorsal anterior agranular insula in rodents has been proposed as part of the rodent equivalent to the primate prefrontal cortex (Markowitsch et al., 1980; Van De Werd and Uylings, 2008). The dorsal agranular insular and ventral anterior insular areas have also been proposed to resemble the primate orbitofrontal cortex (Zilles and Wree, 1995; Dalley et al., 2004). Hence, further research will be required to determine whether the regions of the rat insula studied in the present report correspond only to the insula in primates, or also comprise cortical regions homologous to human prefrontal or orbitofrontal cortices.

## Author Contributions

MM-R contributed to the experimental procedures, analyzed the data, and designed figures. SL contributed to the experimental procedures, coordinated the experiments, analyzed the data, designed figures, and performed the manuscript consistency check. RM-A contributed to the experimental procedures and analyzed the data. DQ-D coordinated the experiments, analyzed the data, and performed the manuscript consistency check. LM and GT contributed to experimental procedures. FC designed experiments, analyzed the data, and performed the manuscript consistency check. RT designed experiments. JS designed and supervised the experiments, wrote the manuscript, and coordinated the study.

## Conflict of Interest Statement

The authors declare that the research was conducted in the absence of any commercial or financial relationships that could be construed as a potential conflict of interest.

## Abbreviations

AMPA: α-amino-3-hydroxy-5-methyl-4-isoxazolepropionic acid
BMI: bicuculline methiodide
cIC: caudal insular cortex
CNQX: cyanquixaline (6-cyano-7-nitroquinoxaline-2,3-dione)
DSM-V: Diagnostic and Statistical Manual of Mental Disorders, Fifth Edition
EPM: elevated plus maze
GABA: γ-aminobutyric acid
GAD: generalized anxiety disorder
gIC: gustatory insular cortex
IC: insular cortex
OCD: obsessive compulsive disorder
pIC: primary interoceptive posterior insucar cortex
PTSD: post-traumatic stress disorder
raIC: rostral agranular insular cotrex
